# Identification of Novel Potential Predisposing Variants in Familial Acute Myeloid Leukemia

**DOI:** 10.1002/cnr2.2141

**Published:** 2024-08-08

**Authors:** Chiara Ronchini, Federica Gigli, Martina Fontanini, Raffaella Furgi, Viviana Amato, Fabio Giglio, Giuliana Gregato, Francesco Bertolini, Michela Rondoni, Francesco Lanza, Atto Billio, Enrico Derenzini, Corrado Tarella, Pier Giuseppe Pelicci, Myriam Alcalay, Elisabetta Todisco

**Affiliations:** ^1^ DIMA Laboratory, Department of Experimental Oncology IEO, European Institute of Oncology IRCCS Milan Italy; ^2^ Onco‐Hematology Division IEO, European Institute of Oncology IRCCS Milan Italy; ^3^ Laboratory of Hematology‐Oncology IEO, European Institute of Oncology IRCCS Milan Italy; ^4^ Hematology Unit and Metropolitan Romagna Transplant Network University of Bologna Ravenna Italy; ^5^ Division of Hematology and Transplant Unit Ospedale di Bolzano Bolzano Italy; ^6^ Department of Health Sciences University of Milan Milan Italy; ^7^ Department of Experimental Oncology IEO, European Institute of Oncology IRCCS Milan Italy; ^8^ Department of Oncology and Hemato‐Oncology University of Milan Milan Italy; ^9^ Ospedale di Busto Arsizio ASST Valle Olona Busto Arsizio Italy

**Keywords:** AML, cancer genetics, cancer predisposition, germline variants

## Abstract

**Background:**

Myeloid neoplasms, including acute myeloid leukemia, have been traditionally among the less investigated cancer types concerning germline predisposition. Indeed, myeloid neoplasms with germline predisposition are challenging to identify because often display similar clinical and morphological characteristics of sporadic cases and have similar age at diagnosis. However, a misidentifications of familiarity in myeloid neoplasms have a critical impact on clinical management both for the carriers and their relatives.

**Aims:**

We conducted a family segregation study, in order to identify novel cancer predisposing genes in myeloid neoplasms and classify novel identified variants.

**Methods and Results:**

We performed a thorough genomic analysis using a large custom gene panel (256 genes), the Myelo‐Panel, targeted on cancer predisposing genes. In particular, we assessed both germline and somatic variants in four families, each with two siblings, who developed hematological neoplasms: seven acute myeloid leukemia and one Philadelphia‐positive chronic myeloid leukemia. In each family, we identified at least one novel potentially predisposing variant, affecting also genes not included in the current European LeukemiaNet guidelines for AML management. Moreover, we suggest reclassification of two germline variants as pathogenic: likely pathogenic p.S21Tfs*139 in *CEPBA* and VUS p.K392Afs*66 in *DDX41*.

**Conclusion:**

We believe that predisposition to hematological neoplasms is still underestimated and particularly difficult to diagnosed. Considering that misidentification of familiarity in myeloid neoplasms have a critical impact on the clinical management both for the carriers and their relatives, our study highlights the importance of revision, in this clinical context, of clinical practices that should include thorough reconstruction of family history and in‐depth genetic testing.

## 1 Introduction

Advent of next‐generation sequencing and large‐scale analysis of mutations in cancer have shown that germline mutations in cancer predisposing genes are more common than previously thought and found in a variable fraction from 5% to 20% of cancer patients, with different prevalence among cancer types. Germline variants are often scored also in sporadic cases of cancer with no familial history [1, 2]. To date, approximately 100 cancer predisposing genes have been associated to hereditary syndromes [3]. The PanCancer analysis scored 8% frequency of pathogenic and likely pathogenic germline mutations in a large cohort of 10 389 patients with 33 different cancer types. Some cancer types show a significant association with mutations in well‐known cancer predisposing genes, that is, ovarian cancer in *BRCA1/2*, whereas new associations were revealed, such as loss of function mutations in *BUB1B* in lung cancer and in *SDHA* in melanoma. These data suggest that a relevant number of cancer predisposing genes and their phenotypic consequences are yet to be identified [4].

Investigation of an increased risk for cancer development has several important clinical implications, both for carriers of the pathogenic variants and their relatives. For cancer patients, identification of a germline pathogenic variant can yield critical information about prognosis and direct therapeutic choices, both in terms of efficiency and toxicity of chemotherapeutic/radiotherapeutic regimens and of surgical intervention strategies. Concerning other family members, identification of pathogenic germline variants in cancer predisposing genes may enhance targeted cancer surveillance and improve cancer prevention [5, 6].

One of the biggest challenges in this field is the classification of the identified germline variants, for two main reasons: (i) most are classified as variants of unknown significance (VUS) and, therefore, have not been assigned a defined biological role nor have they been attributed to particular disease phenotypes [7, 8]. Consequently, such variants have no relevance for clinical applications. (ii) Each cancer predisposing gene may confer different cancer risks, ranging from high to low penetrance (probability) of cancer development, depending both on cancer type and type of variants affecting the genes [9, 10, 11].

Acute myeloid leukemia (AML) is among the malignancies with the lowest frequency of germline variants (4.2% in PanCancer [4]) and traditionally it has been among the less investigated cancer types from this point of view. However, germline predisposition of hematological neoplasms is increasingly assessed and, in recent years, advent of molecular testing has led to identification of specific hereditary hematological syndromes [11, 12, 13]. The current guidelines from the European LeukemiaNet (ELN) for diagnosis and management of AML in adult patients recommend investigation of germline predisposition [14]. This is particularly critical for clinical management of AML patients that often receive allogeneic stem‐cell transplantation to consolidate disease remission. Considering that usually relatives are the best donors, it is critical to exclude the presence of hematopoietic stem cells harboring pathogenic variants from the donor before transplantation [5, 15]. The list of cancer predisposing genes in hematological neoplasms is likely to be largely incomplete and we are in need for further investigations in order to uncover other players of hematological hereditability and define the penetrance of the identified variants.

We designed a comprehensive gene panel for the assessment of germline pathogenic variants in hematological neoplasms, covering the entire coding sequence of 256 genes. The employment of large gene panels enlarges the testing potential compared with genetic screens restricted to high penetrance genes, allowing discovery of more potential predisposing variants; however, it also poses the challenge of assigning a specific functional/phenotypic consequence to newly identified VUS [8]. Variants can also be reclassified when more information on gene function and/or data from family history become available over time, based on new studies. We studied four families with two siblings each affected by myeloid neoplasms in order to exploit the potential of family segregation studies in this context.

## 2 Materials and Methods

### 2.1 Patients

We enrolled four families with history of hematological disorders. Each family comprised two siblings, of which at least one was diagnosed, treated, and/or monitored in our institute. For routine clinical management, patients underwent bone marrow (BM) evaluation including morphological, immunophenotypic and cytogenetic analysis. The main patient characteristics are reported in Table 1. All patients gave written informed consent to their participation to the diagnostic and treatment program, according to the IEO ethical committee approval.

**TABLE 1:** Main clinical features of our families' cohort.

Family	Patient ID	Age at diagnosis	Sex	Disease	Karyotype	Molecular biology^a^	Disease phase (% blasts)	Chemiotherapeutic treatment	Follow‐up
Family1	ID1	17	F	AML	46,XX[20]	Negative	CR (1%)	Induction: mitoxantrone plus cytarabine Consolidation: ‐HD‐ARA‐C, ASP, AMSA ‐6‐MP, ARA‐C	CR
	ID2	32	F	AML	46,XX	NA	/	Induction: scheme 3 + 7 Consolidation: 4 cycles HD‐ARA‐C	CR
Family2	ID3	62	M	AML	46,XY,del(16)(q22qter)[7]/46,XY[13]	JAK2 p.V617F (VAF = 3.55%)	Diagnosis (20%)	Induction: scheme ICE Consolidation: ‐2 cycles HD‐ARA‐C ‐autologous PBSC transplant Reinduction: scheme FLAI Consolidation: 1 cycle HD‐ARA‐C Allogeneic haploidentical PBSC transplant	CR post‐Allo‐TMO
	ID4	53	M	Ph + CML	46,XY,t(9;22)	NA	/	Imatinib; dasatinib; ponatinib	Molecular remission
Family3	ID5	74	M	AML	46,XY[20]	ASXL1 p.R417* (VAF = 10.75%)	Diagnosis (25%)	Induction: ICE scheme Reinduction: FLAI plus Ven scheme Allogeneic haploidentical PBSC transplant Salvage therapy: azacitidine plus Ven	Dead
						TET2 p.L1721Ffs*24 (VAF = 10.83%)			
						TET2 p.D77Tfs*18 (VAF = 21.12%)			
						U2AF1 p.S34F (VAF = 11.5%)			
	ID6	61	M	AML	46,XY,+8	NA	Diagnosis (NA)	Induction: ICE scheme Consolidation: ‐IC scheme ‐1 cycle ARA‐C plus reinfusion of autologous PBSC Allogeneic PBSC transplant from a MUD	Dead
Family4	58	F	AML with myelodisplastic features	46,XX[15]	SF3B1 p.K700E (VAF = 33.7%)	Diagnosis (20%)	Induction: 3 + 7 scheme plus GO Reinduction: ‐FLAI plus Ven scheme ‐Azacitidine plus Ven 1 cycle Allogeneic haploidentical PBSC transplant	Dead
					TET2 p.T229Nfs*25 (VAF = 32.93%)			
					TET2 p.E566* (VAF = 31.32%)			
					ETV6 p.V158Pfs*10 (VAF = 17.87%)			
	ID8	73	M	AML with myelodisplastic features	46,XY[20]	IDH2 p.R140Q	Diagnosis (15%–20%)	Azacitidine plus Ven 8 cycles	CR

Abbreviations: 3 + 7, cytarabine+daunorubicin; 6‐MP, mercaptopurine; Allo‐HSCT, allogeneic hematopoietic stem‐cell transplantation; AML, acute myeloid leukemia; AMSA, amsacrine; ARA‐C, cytarabine; ASP, asparaginase; CR, complete remission; GO, gentuzumab ozogamicin; HD, high dose; IC: idarubicine, cytarabine; ICE: idarubicine, cytarabine and etoposide; MRD, minimal residual disease; MUD, matched unrelated donor; NA, not available; PBSC, peripheral‐blood stem cells; Ph + CML, Philadelphia‐positive (Ph+) chronic myeloid leukemia; VAF, variant allele frequency; Ven, venetoclax.

^a^ Next generation sequencing analysis of PB DNA using the Oncomine Myeloid Research Assay and the Ion Torrent S5 technology (ThermoFisher).

### 2.2 Molecular Analyses

We extracted DNA from BM aspirates or peripheral blood draws for the analysis of somatic variants, and from buccal swabs for the analysis of germline variants, using the QIAamp DNA mini kit following the manufacturer's instructions (Qiagen). As previously described [16], we performed two genomic characterizations: one for routine clinical practice and one for research purposes. For clinical assessment, we used a commercially available diagnostic NGS assay, the Oncomine Myeloid Research Assay (40 key DNA target genes and 29 driver genes in a broad fusion panel). Data analysis was performed with IonReporter software applying the last release of myeloid workflow (ThermoFisher Scientific). For research purposes, we used the Myelo‐panel, a custom gene panel for analysis of 256 cancer predisposing genes, purposely designed to stratify patients for possible targeted therapies and to identify germline variants associated with predisposition to develop myeloid neoplasms. The 256 cancer predisposing genes include: 79 susceptibility genes, the most frequently associated with risk of development of hematological tumors; 38 AML drivers, with a key role in leukemogenesis; 113 actionable genes, with a concrete clinical manageability; 26 genes belonging to more than one of these categories and 126 pharmacogenomics single‐nucleotide polymorphisms (SNPs), allelic variants associated with susceptibility to certain drugs (see Table S1 for gene list).

Library preparation was performed using Ion Torrent Ion AmpliSeq Library Kit 2.0 and libraries were sequenced with Ion S5 system according to the manufacturer's recommendations (ThermoFisher Scientific), reaching a median sequencing coverage of ~1300X. Variant analysis was performed with Ion Reporter software and filtering as previously described [16]. In details, for identification of somatic variants in the tumoral lesions, first of all, in order to subtract each individual germline variants, we employed buccal DNA samples as normal reference for each patient. Moreover, we selected: (i) variants in exonic regions of the genome and affecting the coding sequence: nonsynonymous, stop‐gain, and stop/start loss SNVs, in‐frame and frameshift indels; (ii) variants with VAF of the alternative allele <40%; (iii) finally, in order to filter for germline polymorphisms, we removed any variant reported in any population database (ESP6500, ExAC, gnomAD, 1000 Genomes Phase 3, dbSNP) with a frequency >0.005.

From buccal DNA, we selected germline variants as: (i) variants in exonic regions of the genome and affecting the coding sequence: nonsynonymous, stop‐gain, and stop/start loss SNVs, in‐frame and frameshift indels; (ii) variants with allelic depth (AD, reads supporting the alternative allele) ≥5 reads and with VAF of the alternative allele ≥20%; (iii) finally, in order to filter for germline polymorphisms, we removed any variant reported in any population database (ESP6500, ExAC, gnomAD, 1000 Genomes Phase 3, dbSNP) with a frequency >0.005.

Variants passing our filters were annotated in terms of pathogenicity using different computational tools, including ClinVar, CancerVar, Intervar, Varsome, and RENOVO [17].

## 3 Results

### 3.1 Clinical History

We identified four families, each with two siblings, who developed hematological neoplasms. Of these eight patients, seven (87.5%) developed acute myeloid leukemias (AML; see Table 1 for clinical details). Patient ID4 of Family2 developed a Philadelphia‐positive chronic myeloid leukemia (Ph + CML). The detailed clinical history of each family is described below.

#### 3.1.1 Family1

Sister ID1 was diagnosed with hyperleukocytotic AML with normal karyotype (NK) at 17 years of age. She received a regimen (LAME 89 pilot study) including an intensive induction phase (mitoxantrone plus cytarabine) and obtained complete remission. She then received two consolidation courses: the first containing timed‐sequential high‐dose cytarabine, asparaginase, and amsacrine; the second consisting of mercaptopurine plus cytarabine for 18 months. She is now 40 years old and still in complete remission (CR).

Sister ID2 also received a diagnosis of AML with NK when she was 32 years old. She obtained CR following induction therapy with the 3 + 7 schedule (cytarabine 100 mg/mq G1, daunorubicin 60 mg/mq G1‐3‐5). She then received four consolidation therapy courses with high doses of cytarabine. After 2 years from end of treatment, she is persistently in CR. Both sisters are currently under close clinical monitoring.

#### 3.1.2 Family2

Brother ID3 developed AML with karyotype 46,XY,del(16)(q22qter)[7]/46,XY[13] at age 62. He received ICE (idarubicine, cytarabine, and etoposide) as induction chemotherapy and achieved CR. He then received a consolidation cycle with high‐dose cytarabine, and peripheral‐blood stem cells (PBSC) were harvested during recovery. He underwent autologous PBSC transplantation as final consolidation therapy, but relapsed a year later. Molecular evaluation identified a known *JAK2 V617F* mutation (Table 1) and additional mutations in the *ASXL1* (p.G1185Cfs*4, varian allele frequency [VAF] = 3.09%) and *RUNX1* (p.L405Cfs*, VAF = 4.71%) genes (not shown). The patient, therefore, received reinduction chemotherapy with FLAI (fludarabine, idarubicine, and cytarabine) and a consolidation cycle with high‐dose cytarabine, achieving CR. Four months later he underwent myeloablative allogeneic haploidentical stem‐cell transplantation from a family donor (conditioning: fludarabine, cyclophosphamide, total body irradiation, and cyclophosphamide +3 and +4 post‐reinfusion), complicated by occurrence of veno‐occlusive disease, which was treated and resolved with defibrotide. Approximately 1 year and 9 months after transplantation, he is in CR in fair general condition.

Brother ID4 was diagnosed with Ph + CML, Sokal score low risk, in absence of additional cytogenetic alterations at age 53. He started therapy with imatinib but immediately developed intolerance and, therefore, changed to dasatinib (tyrosine kinase inhibitor—TKI—secound generation), achieving complete molecular response. Three years after starting therapy, he developed laterocervical lymphadenopathy and persistent fever; he was deemed intolerant to dasatinib, which was discontinued. He then started therapy with ponatinib (third generation TKI), with persistence of molecular remission to date.

#### 3.1.3 Family3

**FIGURE 1:**
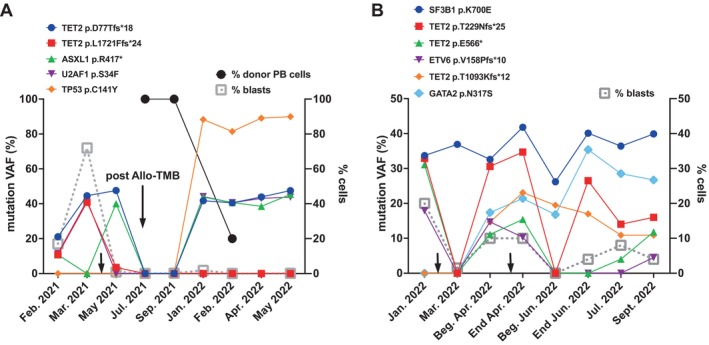
Evolution of the genomic landscape in two patients of our cohort. For each patient, we show the variant allele frequency (VAF, left *Y*‐axis) of each somatic mutation identified by clinical monitoring with the NGS Oncomine Myeloid Research Assay. Each mutation is labeled according to the left legend shown above the graphs. On the *X*‐axis we report the date of testing. Response to treatment was monitored in the BM of the patient by immunophenotype of the myeloid blasts (% blasts, right *Y*‐axis). (A). Patient ID5. In panel A, we also show the percentage of donor cells in the peripheral blood (% donor PB cells, right *Y*‐axis), following allogeneic BM transplantation (post Allo‐TMB). The small black arrow indicates the timing of induction chemotherapy. (B). Patient ID7. The first black arrow shows the timing of induction therapy and the second of re‐induction therapy. Beg., beginning.

Brother ID5 was diagnosed with AML characterized by myelodysplastic changes, NK and mutations in *TET2, ASXL1*, and *U2AF1* genes (Table 1) at age 74. He was treated with ICE to which the patient was refractory. Therefore, reinduction chemotherapy according to FLAI plus venetoclax was performed, resulting in CR. Four months later, he underwent myeloablative haploidentical allogeneic transplantation (conditioning as for brother ID3), maintaining CR and achieving complete full chimera. Seven months after transplantation, he showed reduction in chimerism with worsening of blood counts and progressive need for transfusion support. Subsequent BM evaluation showed no increase in blasts, a progressive increase in VAF of *TET2*, *ASXL1*, and *U2AF1* mutations and acquisition of a *TP53* mutation (Figure 1A). These clinical and molecular parameters were considered indicative of disease relapse. The patient was then treated with salvage therapy (azacitidine + venetoclax) followed by worsening of his general condition and progressive leukocytosis; BM evaluation showed refractoriness of the disease with transformation into megakaryoblastic AML. The patient died of disease progression approximately 1 year after transplantation.

Brother ID6 was diagnosed with AML bearing trisomy of chromosome 8 at age 61. He received induction chemotherapy with ICE achieving CR. He received further cycles of IC (idarubicin and cytarabine) as consolidation and cycle A8 (cytarabine) followed by reinfusion of autologous PBSCs. When in chronic remission, he underwent allogeneic myeloablative transplantation from a volunteer donor (conditioning: busulfan, fludarabine, ATG). Post‐transplantation recovery, he was undermined by a state of immunosuppression, showing cytomegalovirus (CMV) reactivation resistant to antiviral treatments, which resulted in graft failure (requiring a boost of PBSCs from the same donor), gram‐positive encephalitis and finally Geotrichum sepsis, which led to patient's death approximately 10 years after transplantation.

#### 3.1.4 Family4

Sister ID7 received diagnosis of AML with myelodysplastic changes and NK when she was 58 years old. NGS mutational analysis of blasts for clinical assessment showed presence of mutations in *SF3B1*, *TET2*, and *ETV6* (Table 1). She received induction therapy with 3 + 7 scheme plus gentuzumab ozogamicin (as part of the GIMEMA 1819 protocol). However, she did not obtain remission and underwent a cycle of reinduction according to FLAI plus venetoclax, obtaining only partial response with persistence of the *SF3B1*, *TET2*, and *ETV6* mutations (Figure 1B). Within 1 month, the disease relapsed and she underwent a further cycle of therapy with azacitidine plus venetoclax, obtaining complete response. She then underwent haploidentical allogeneic transplantation (conditioning with treosulfan + fludarabine). Nine days after, she had an acute episode of cerebral hemorrhage, which caused her death.

Brother ID8 was diagnosed at age 73 with AML displaying myelodysplastic changes, NK and presence of the hotspot *IDH2* mutation (Table 1). He achieved CR after the first course of azacitidine plus venetoclax therapy. To date, he underwent eight chemotherapeutic cycles, maintaining CR. The favorable outcome observed in this patient is in line with the results of a recent clinical trial showing that the combination of azacitidine plus venetoclax in patients with mutations in *IDH1/2*, and in *IDH2* in particular, have high response rates, durable remissions, and significant OS [18].

### 3.2 Germline and Somatic Mutational Analysis

To define if the patients described above had germline predisposition to developing myeloid neoplasms and, when possible, define the somatic landscapes of their malignancies, we performed mutational analyses using the Ion Torrent technology and a custom gene panel designed in our laboratory, the Myelo‐panel (see Materials and Methods for details).

We performed germline analysis on buccal DNA and, whenever available, somatic analysis on the pathological sample at diagnosis, as previously described [16]. We report all germline variants (VAF >20%) identified in common between the sibling pairs (Table 2) and, for somatic mutations, all pathogenic and likely pathogenic variants plus VUS identified with VAF >5% (Table 3). All germline variants were validated by Sanger sequencing (Figure S1) and the family trees of our families are shown in Figures S3–S5.

**TABLE 2:** Germline variants identified in our cohort.

Family	Patient ID	GENE	Transcript	Nucleotide change	AA change	Variant Classification^a^	VAF (%)
Family1	ID1	*CEBPA*	NM_004364.4	c.62delG	p.S21Tfs*139	Likely pathogenic	51.97
	ID2	*CEBPA*	NM_004364.4	c.62delG	p.S21Tfs*139	Likely pathogenic	50.84
Family2	ID3	*DDX41*	NM_016222.4	c.1174_1175delAA	p.K392Afs*66	VUS	51.66
	ID4	*DDX41*	NM_016222.4	c.1174_1175delAA	p.K392Afs*66	VUS	48.64
Family3	ID5	*LYST*	NM_001301365.1	c.10193G > A	p.R3398Q	VUS	51.83
	ID6	*LYST*	NM_001301365.1	c.10193G > A	p.R3398Q	VUS	51.96
Family4	ID7	*FANCA*	NM_000135.4	c.2574C > G	p.S858R	VUS (pathogenic in FAMutdb)	42.39
		*JAK2*	NM_004972.4	c.1711G > A	p.G571S	VUS	47.95
		*ERBB4*	NM_005235.3	c.3838G > C	p.E1280Q	VUS	49.79
	ID8	*FANCA*	NM_000135.4	c.2574C > G	p.S858R	VUS (pathogenic in FAMutdb)	45.15
		*JAK2*	NM_004972.4	c.1711G > A	p.G571S	VUS	47.95
		*ERBB4*	NM_005235.3	c.3838G > C	p.E1280Q	VUS	48.41

Abbreviations: AA change, aminoacid change; FAMutdb, Fanconi Anemia Mutation Database; VAF, variant allele frequency; VUS, variant of unknown significance.

^a^ Laboratory classification based on annotation in ClinVar, Varsome, and Intervar.

**TABLE 3:** Somatic variants identified in our cohort.

Family	Patient ID	Gene	Transcript	Nucleotide change	AA change	Variant classification^a^	VAF (%)
Family1	ID1	None	/	/	/	/	/
	ID2	NA	/	/	/	/	/
Family2	ID3	*DDX41*	NM_016222.4	c.1574G > A	p.R525H	Pathogenic	7.95
		*JAK2*	NM_004972.3	c.1849G > T	p.V617F^b^	Pathogenic	3.56
		*DNMT3A*	NM_175629.2	c.2330C > A	p.P777H	Likely pathogenic	10.58
	ID4	NA	/	/	/	/	/
Family3	ID5	*ASXL1*	NM_015338.6	c.1249C > T	p.R417*^b^	Pathogenic	13.88
		*RAD50*	NM_005732.4	c.2801delA	p.N934Ifs*6	Pathogenic	13.16
		*TET2*	NM_001127208.2	c.5163delG	p.L1721Ffs*24^b^	Likely pathogenic	17.83
		*IGFN1*	NM_001164586.2	c.6746A > G	p.D2249G	VUS	12.64
	ID6	*IDH2*	NM_002168.4	c.515G > A	p.R172K	Pathogenic	36.86
Family4	ID7	*SF3B1*	NM_012433.3	c.2098A > G	p.K700E^b^	Pathogenic	34.19
		*TET2*	NM_001127208.2	c.1696G > T	p.E566*^b^	Likely pathogenic	29.56
		*ETV6*	NM_001987.4	c.472_473delGT	p.V158Pfs*10^b^	Likely pathogenic	16.59
		*GATA2*	NM_032638.4	c.968A > C	p.H323P	VUS	31.71
		*FGFR3*	NM_000142.4	c.1900G > A	p.A634T	VUS	5.35
		*BRCA2*	NM_000059.3	c.4634 T > C	p.L1545P	VUS	5.09
	ID8	*SF3B1*	NM_012433.3	c.2098A > G	p.K700E	Pathogenic	31.74
		*IDH2*	NM_002168.3	c.419G > A	p.R140Q^b^	Pathogenic	11.64
		*IDH1*	NM_005896.3	c.395G > A	p.R132H	Pathogenic	2.38

Abbreviations: AA change, aminoacid change; NA, not available; VAF, variant allele frequency; VUS, variant of unknown clinical significance.

^a^ Laboratory classification based on annotation in ClinVar and CancerVar.

^b^ Variants found in common with clinical assessment of mutational analysis (see Table 1).

#### 3.2.1 Families With Mutations in Genes Reported in Hereditary Hematologic Malignancy Syndromes

In Family1 and Family2 of our cohort, we identified germline mutations in two genes known to confer inherited risk to development of MDS/AML: *CEBPA* and *DDX41*. In particular, both sisters in Family1 harbored a likely pathogenic indel p.S21Tfs*139 in *CEBPA*. The mutation is heterozygous in both sisters with VAF = 51.9% and 50.8%, respectively (Table 2). As expected for CEBPA‐associated familial AML, both sisters developed their disease at very young age (Table 1). We performed somatic mutational analysis only for sister ID1 and detected no somatic mutations (Table 3). However, this result may not be significant since BM aspirate was collected when she was in CR and immunophenotypic analysis used for NGS showed less than 1% blast infiltration.

In Family2 both brothers had the germline heterozygous frameshift indel p.K392Afs*66 in *DDX41*, with VAF = 51.7% and 48.6%, respectively. This variant is currently annotated as VUS (Table 2). For this family, we had tumor DNA available only for one brother. As often reported for *DDX41*‐associated familial AML, patient ID3 harbored also the hotspot p.R525H somatic mutation for *DDX41* (VAF = 7.95%), together with two other somatic mutations: the most common pathogenic variant in *JAK2* p.V617F (VAF = 3.56%) and the likely pathogenic mutation p.P777H in *DNMT3A* (VAF = 10.58%; Table 3).

#### 3.2.2 Family With a Germline Mutation in the LYST Gene

In Family3 both brothers displayed a germline missense single‐nucleotide mutation in the *LYST* gene, translating in p.R3398Q (Table 2). This variant is currently annotated as VUS and the functional consequences are unknown. Both siblings developed AML and somatic mutational analysis of their leukemic samples at diagnosis identified pathogenic variants. Brother ID5 harbored truncating pathogenic indels in *ASXL1* (p.R417*) and in *RAD50* (p.N934Ifs*6), a likely pathogenic truncating indel in *TET2* (p.L1721Ffs*24) and a VUS in *IGFN1* (p.D2249G). Brother ID6 harbored the second most common somatic pathogenic variant in *IDH2*, p.R172K, with VAF = 36.9% (Table 3).

In order to reconstruct the genomic evolution of the disease in patient ID5, we exploited NGS analysis performed in our Institute on BM aspirates for clinical monitoring using the commercially available Oncomine Myeloid Research Assay. As shown in Figure 1A, there was partial response to treatment post‐induction with disappearance of cells bearing *TET2* p.L1721Ffs*24 and *U2AF1* p.S34F variants, but there were no effects on the blasts harboring *ASXL1* p.R417* and *TET2* p.D77Tfs*18 mutations. The patient underwent CR post‐allo TMB, however, unfortunately, he relapsed very rapidly with re‐expansion of the same AML clone detected pre‐transplant and appearance of a new mutation in *TP53*, p.C141Y, that reached a VAF around 90%. The patient succumbed of his disease less than 8 months after relapse.

#### 3.2.3 Family With a Complex Germline Mutational Landscape

In Family4, both siblings carried three heterozygous germline mutations annotated as VUS: p.S858R in *FANCA*, p.G571S in *JAK2* and p.E1280Q in *ERBB4* (Table 2). These patients also shared, in their AML samples at diagnosis, the presence of the most common somatic pathogenic variant in *SF3B1*: p.K700E, with a VAF of 34.2% and 31.7% in patient ID7 and ID8, respectively (Table 3). Using Sanger sequencing, we confirmed this is an acquired somatic mutation in a hematopoietic clone with high VAF; indeed, the variant was not detected in buccal DNA collected from either siblings (Figure S2). Patient ID7 harbored also two likely pathogenic somatic variants (*TET2* p.E566* and *ETV6* p.V158Pfs*10) and 3 VUS with VAF >5% (*GATA2* p.H323P, *FGFR3* p.A634T and *BRCA2* p.L1545P; Table 3). Patient ID8 harbored two subclonal pathogenic mutations in both *IDH2* (p.R140Q; VAF = 11.64%) and *IDH1* (p.R132H; VAF = 2.38%).

As for patient ID5, we were able to reconstruct the genomic evolution of AML in patient ID7, based on NGS analysis performed for clinical monitoring. Following induction chemotherapy, the patient showed persistence of the hematopoietic clone harboring the *SF3B1* p.K700E mutation, which maintained almost constant VAF (30%–40%), showing complete resistance along the entire course of the disease. After induction, the blasts acquired two new variants: *TET2* p.T1093Kfs*12 and *GATA2* p.N317S. Therefore, the patient underwent re‐induction chemotherapy, that affected the primary responsive clone, but again showed no efficacy on the clone with *SF3B1* p.K700E variant or clones with the two mutations acquired post‐induction. This patient succumbed to the disease within 3 months post‐relapse, with reappearance of all mutations identified both pre and post‐induction therapy (Figure 1B).

## 4 Discussion

We report the results of detailed genomics studies on four families in which pairs of siblings developed myeloid neoplasms and, in particular, AML in seven out of eight cases. Each family was characterized by a unique genomic landscape, both at the germinal and somatic levels, with no mutations in common among the four different families. This heterogeneity was also mirrored by independent clinical histories and responses to therapies and survival of our patients. This is in line with the extremely heterogenous landscape, both intra and inter‐patients, that characterize hematological neoplasms [19]. Moreover, AML is a clonal disorder, in which heterogeneity is supported by many genetically distinct clones that coexist in the same individual and may evolve through different routes. Indeed, the clonal composition of AML continually evolves thanks to the complex and dynamic interplay of emergence of new genetic aberrations and the selective pressures of intrinsic and extrinsic factors, such as chemotherapeutic treatments [19]. For two of our patients, we observed these heterogeneity and dynamic clonal evolution during treatment, with some clones resistant to treatment and appearance of new genetic variants at relapse, in both patients (Figure 1).

According to the current World Health Organization (WHO) classification of hematological neoplasms with germline predisposition and the last published recommendations from ELN for diagnosis and management of AML, we identified germline mutations both in genes with a well‐established role in predisposing to the development of MDS/AML and genes with no defined role in this context [14, 20]. Indeed, *CEBPA* and *DDX41* are included in myeloid neoplasms with germline predisposition without a pre‐existing platelet disorder; *FANCA* is listed in the category of genes mutated in myeloid neoplasms associated with bone marrow failure syndromes; *JAK2* is included in emerging disorders with germline predisposition; and, finally, *LYST* and *ERBB4* have not been reported yet in such classifications. None of the identified mutations is currently annotated as pathogenic in cancer databases; however, they could all play an important role in hematological diseases development.

To the best of our knowledge, the specific variants in *CEBPA* and *DDX41* identified in our study have not been described before in AML cases. However, the variant identified in *CEBPA* is a truncating mutation and, importantly, it is located at the 5′‐end of the gene (c.62, p.S21). N‐terminal mutations in *CEBPA* are known to have a penetrance close to 100% of leukemia development; however, they correlate with a favorable prognosis [21]. Because of the unavailability of a sample at diagnosis, we could not assess if in our patients there was acquisition of a somatic *CEPBA* mutation; nonetheless, both siblings are currently in CR and under clinical monitoring. The frameshift indel identified in *DDX41* tumor suppressor gene is currently annotated as VUS and it is not listed either in COSMIC or cBioportal. This mutation is expected to truncate the protein before its functional helicase domain and likely causes a loss of function. Truncating mutations in *DDX41* have been shown to increase the risk of developing myeloid neoplasms and are associated with faster progression to leukemia [22, 23]. Both our patients are males who, in presence of *DDX41* mutations, are expected to develop myeloid malignancies more frequently than females [22, 23]. Moreover, patient ID3 harbored the hotspot mutation p.R525H in *DDX41*, frequently acquired as somatic mutation in carriers of germline *DDX41* variants [24, 25]. No pathological DNA was available for the other sibling, who however developed a Ph + CML.

The *LYST* gene is mutated in autosomal recessive mode in inborn errors of immunity syndromes and, in particular, in familial hemophagocytic lymphohistiocytosis (FHL) syndromes with hypopigmentation: Chediak–Higashi syndrome and hemophagocytic lymphohistiocytosis (HLH) [26]. LYST is a lysosomal trafficking regulator and a key effector of cytotoxic granules' biogenesis. It is involved in the modulation of cytotoxic T‐lymphocytes (CTL) and natural killer (NK)‐cell functions by regulating degranulation. LYST‐deficient CTLs and NK‐cells display impaired ability to kill target cells and accumulate giant cytotoxic granules [27, 28]. Patients with Chediak–Higashi syndrome display oculocutaneous albinism, easy bruising, recurrent pyogenic infections, and exhibit abnormal functions of NK‐cells and alterations in neutrophils, leading to neutropenia [28]. The variant we identified has been reported as germline in a case affected by Chediak–Higashi syndrome. We have no evidence to infer a direct role for the identified *LYST* mutation in predisposing to development of myeloid neoplasms; however, we can envision a possible role for a deregulated immune system in controlling the homeostasis of hematopoietic differentiation.

In the fourth family we scored a more complex landscape with three germline VUS shared by both siblings. The most noteworthy is mutation p.S858R in *FANCA* gene. It is annotated as VUS in cancer databases; however, notably, it is annotated as pathogenic in FAMutdb, a database of variants identified in Fanconi Anemia (FA) (http://www2.rockefeller.edu/fanconi/) and it is reported in several FA patients of different origins (Italian, German, Indian‐Jewish) [29, 30, 31]. In particular, within the Indian‐Jewish cohort, two children with the *FANCA* p.S858R variant developed AML at very young age (7 and 10 years old) [31]. FA is an autosomal recessive disease usually associated to mutations of other *FANC* members for its manifestation. The penetrance and phenotypic manifestations of the syndrome are highly variable [32]. Our patients had no signs of FA and we did not identify pathogenic mutations in other sequenced members of *FANC* gene family. However, carriers of heterozygous FA mutations present increased risk for development of MDS and AML [33]. Therefore, we can envision an incompletely penetrant phenotype imposed by p.S858R *FANCA* mutation, which requires cooperation with other germline lesions.

Germline predisposition to myeloid neoplasms due to pathogenic or likely pathogenic variants of *JAK2* gene are emerging as new disorders; however, in both siblings of Family4, we identified a germline variant currently annotated as VUS: p.G571S. This mutation is located in exon 13 and, as the most common oncogenic variant in *JAK2*, p.V617F, within the region encoding for the autoinhibitory JH2 pseudokinase domain of the protein. Although the biological significance of this variant is not well established yet, in vitro assays in Ba/F3 cells suggest no significant impact on the *JAK2* protein functions [34]. This variant has been reported associated to MPN with a frequency of around 0.01% and as germline both in sporadic and in familial cases of essential thrombocythemia (ET) [35, 36]. Moreover, it is listed in COSMIC (COSM29107, COSM142855: 10 mutations, seven of which in the hematopoietic and lymphoid category).

Finally, the missense variant in the receptor tyrosine kinase *ERBB4* has an unknown biological significance and it has not been reported in COSMIC. We currently have no clues on its possible role in our clinical context.

Although functional characterization of these mutations is still required, we speculate that in Family4 the full MDS/AML phenotype may result from the cooperation between the *FANCA* and *JAK2* missense mutations. Interestingly, in a WES analysis of mutations in a cohort of patients with BM failure syndromes of suspected inherited origin, 11.6% of patients carried two co‐occuring potential alterations [37].

In Family4, in siblings sharing this same complex germline landscape, the clinical history of the disease followed independent pathways, with acquisition of independent somatic mutations except for a common prevalent clone (VAF >30% in both siblings) with p.K700E mutation in *SF3B1*, suggesting a selective pressure imposed by the germline variants on acquisition of this somatic mutation. In a cohort of 16 FA patients, this same mutation in *SF3B1* was identified in a patient with a germline *FANCA* mutation, who developed refractory anemia with ring sideroblasts and mutations in *SF3B1* and *JAK2* seem to co‐occur in myelodysplastic/myeloproliferative neoplasms with ring sideroblasts and thrombocytosis [38, 39]. Notably, clonal evolution reconstruction by single‐cell sequencing in a FA patient harboring a mutation in *FANCA*, showed appearance, in the early stage of MDS, of a clone with the *SF3B1* p.K700E mutation, which expanded to become dominant with progression of the disease to AML, together with a mutation in *RUNX1* [40].

We think that genetic testing for the mutations we identified in the relatives of our families should be mandatory and could be instrumental in driving clinical decisions. The presence of germline mutations in *DDX41* or *CEBPA* could inform clinicians about the relative risks of developing hematological tumors, the possible age at onset and the prognosis of the possible disease. Upon possible identification of these variants, clinicians could plan ad hoc clinical monitoring for early detection of the disease, even in absence of symptoms, and treatment strategies, avoiding excessive medical testing, and useless overtreatment. Indeed, the presence of the *CEBPA* mutation clearly put at high risk (close to 100%) of developing AML at very young age (median age ~20 years old); however, the prognosis is usually good. Conversely, the presence of the mutation in *DDX41* puts individuals at lower risk of developing hematological neoplasms, which, however, display onset at much older age (very rarely before 40 years old and with a median age between 60 and 70 years) and are categorized as high risk. Furthermore, in Family4, we observed a convergent evolution of the disease in the two siblings. Our data suggest, in particular, that the presence of a germline mutation in the *FANCA* gene could favor the acquisition and/or the expansion of a hematological clone harboring the *SF3B1* p.K700E mutation. This should instruct caretakers in being particularly careful in collecting clinical information and reconstructing family histories in cases of AML harboring the somatic mutation *SF3B1* p.K700E and planning for eventual germline genetic testing for *FANCA*.

Currently, a field of intense investigation is the study of clonal hematopoiesis (CH), a premalignant state in which hematopoietic stem and progenitor cells clonally expand due to acquisition of somatic mutations in genes that confer selective growth advantages. CH has been associated to increase risk of development of a number of diseases that include both hematological tumors and nonmalignant conditions such as ischemic cardiovascular, inflammatory and autoimmune diseases. The analysis of CH is performed on peripheral blood in several clinical contexts, independent from hematological disease, by NGS that include both WES and targeted gene panels [41]. The search for CH in an ever‐growing number of nonhematological patients will generate a critical mass of genomic data on the hematopoietic system that could prove extremely helpful in the field of germline predisposition to myeloid neoplasms, significantly increasing the wealth of information available for this traditionally under investigated cancer type.

We acknowledge that we analyzed a relatively small cohort. Nonetheless, we selected families with siblings with a strong malignant hematological phenotype. This allowed us to perform genotype/phenotype associations and to draw quite solid conclusions on the impact of specific mutations. Indeed, according to The American College of Medical Genetics and Genomics (ACMG) guidelines interpretation of sequencing variants [42], a critical component for understanding of significance of a VUS is the observed clinical phenotype. On these basis, we suggest reclassification as pathogenic of the likely pathogenic p.S21Tfs*139 in *CEPBA* and the VUS p.K392Afs*66 in *DDX41*. Moreover, our study underlines the invaluable relevance of testing multiple affected relatives belonging to the same family, in order to discover potential novel hereditary hematological syndromes. Importantly, we show that familiarity in hematological tumor is definitively more common than currently thought and that with thorough genomic analysis using large gene panels appositively targeted on cancer predisposing genes, it is possible to identify novel germline variants in unsuspected cases. Indeed, based exclusively on their clinical parameters, none of the cases of our cohort would have been suspected of familiarity. However, in each of our families, we identified at least one novel variant, affecting also genes not included in the current ENL guidelines for AML management. Finally, considering that myeloid neoplasms with germline predisposition often display clinical and morphological characteristics similar to sporadic cases and that age at diagnosis in the two groups often overlap, it is really challenging to suspect familiarity in the context of hematological tumours [43]. However, studying affected siblings, we identified potential germline predisposition in each family. Our data underline how current clinical practice underestimate familial cases within hematological neoplasms and calls for implementation of novel clinical practices that should include thorough reconstruction of personal and family history and genetic testing with large gene panels targeted for predisposing genes.

## Author Contributions

**Chiara Ronchini:** supervision (lead), writing – original draft (lead), writing – review and editing (lead), visualization (lead), investigation (supporting), formal analysis (supporting). **Federica Gigli:** resources (lead), writing – original draft (supporting). **Martina Fontanini:** investigation (equal), formal analysis (equal). **Raffaella Furgi:** investigation (equal), formal analysis (equal). **Viviana Amato:** resources (supporting). **Fabio Giglio:** resources (supporting). **Giuliana Gregato:** resources (supporting), investigation (supporting). **Francesco Bertolini:** resources (supporting), investigation (supporting). **Michela Rondoni:** resources (supporting). **Francesco Lanza:** resources (supporting). **Atto Billio:** resources (supporting). **Enrico Derenzini:** resources (supporting), project administration (supporting). **Corrado Tarella:** resources (supporting), project administration (supporting). **Pier Giuseppe Pelicci:** supervision (supporting), funding acquisition (supporting). **Myriam Alcalay:** funding acquisition (lead), Conceptualization (equal), supervision (supporting), project administration (supporting), writing – review and editing (supporting). **Elisabetta Todisco:** Conceptualization (equal), supervision (supporting), funding acquisition (supporting), project administration (lead), writing – review and editing (supporting).

## Conflicts of Interest

The authors declare no conflicts of interest.

## Supporting information


Data S1.

Table S1.

## Data Availability

The data that support the findings of this study are available from the corresponding author upon reasonable request.
