# Supplementary material for: Identification of Novel Potential Predisposing Variants in Familial Acute Myeloid Leukemia
Source: Cancer Rep (Hoboken). 2024 Aug 8;7(8):e2141. doi: 10.1002/cnr2.2141 (PMC11310090; doi:10.1002/cnr2.2141)
Supplement: Supplementary file 1 — Data S1. [file CNR2-7-e2141-s002.pdf]

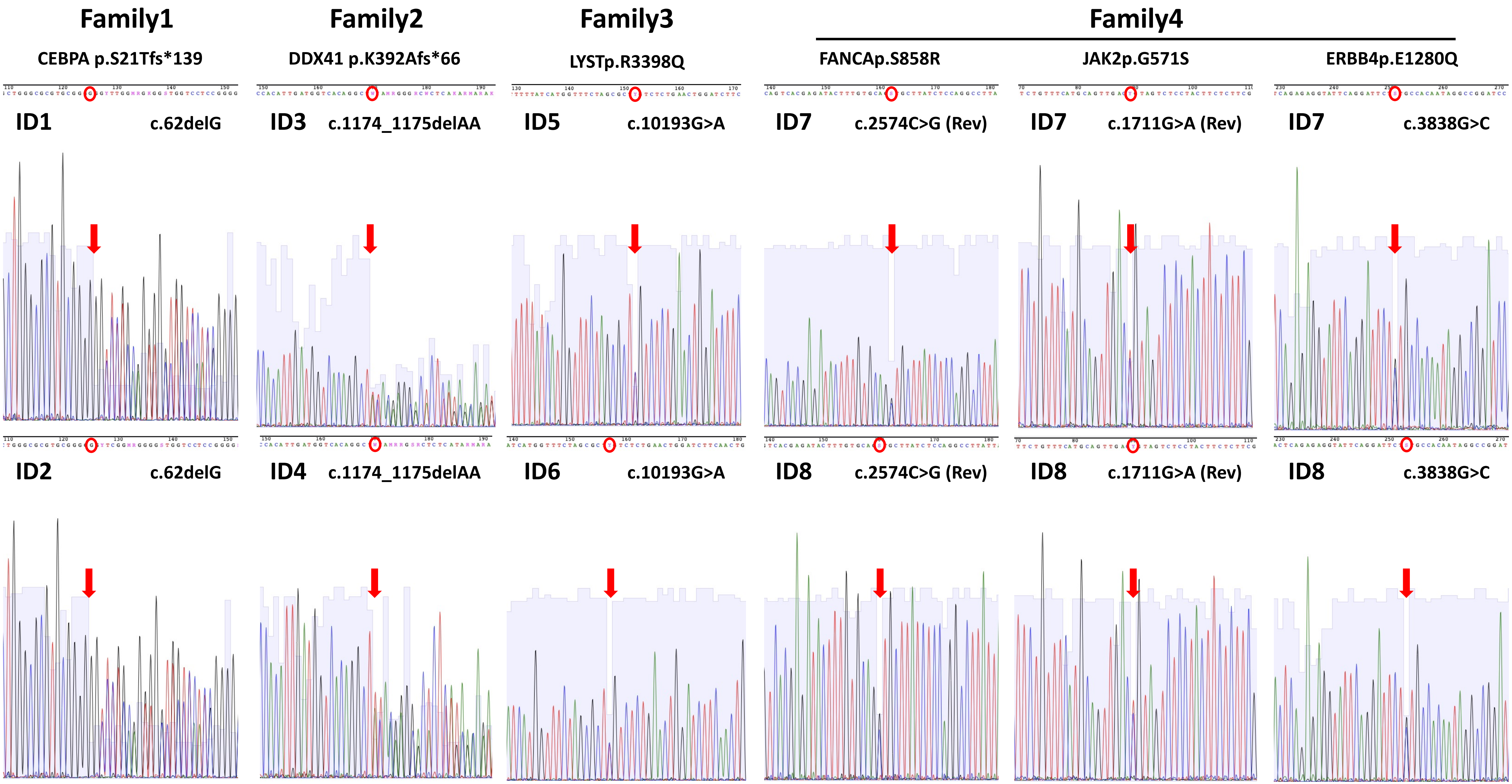

Supplementary Figure 1. Validation by Sanger sequencing of the germline mutations identified in our families. Direct sequencing electropherograms of the germline genetic variants identified in the families included in our study. For each family is indicated each tested patient (ID#) and each tested variant: aminoacidic change at the top of the figure (p.) and the nucleotide change (c.) in the figure. The red arrow in each figure points to the mutated position and the mutated nucleotide is circled in red. Rev, reverse.

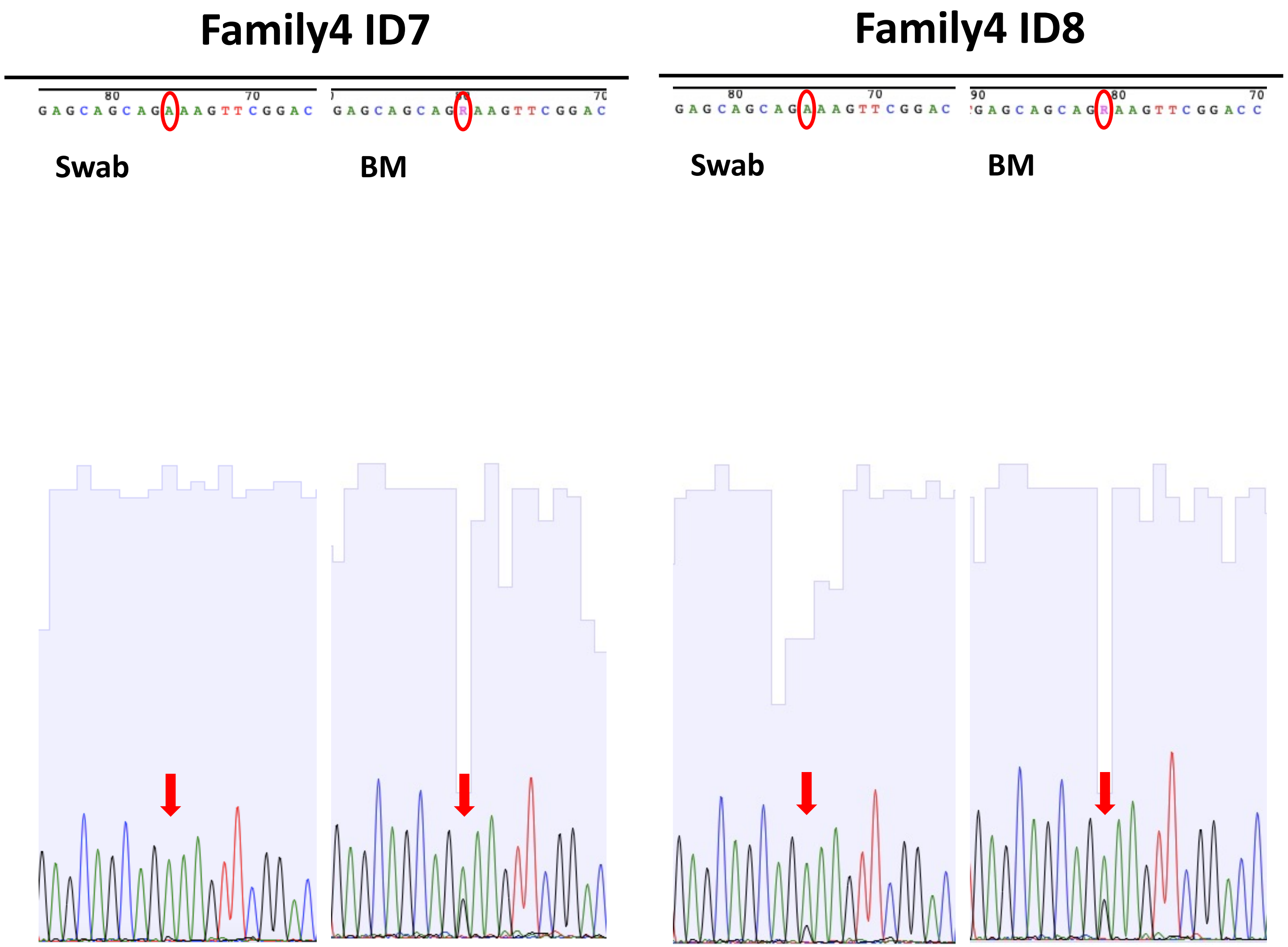

Supplementary Figure 2. SF3B1 p.K700E is a somatic variant in Family4. Direct sequencing electropherograms of the variant p.K700E (c.2098A>G) identified in SF3B1 in both siblings of Family4. For both patients (ID7 and ID8), we tested DNA extracted from both buccal swab (Swab) and bone marrow mononuclear cells (BM). The red arrow in each figure points to the mutated position and the mutated nucleotide is circled in red. In patient ID8 is visible a minor contamination by PB mutant cells in the buccal swab (VAF<5%). We are showing the reverse and complement of the forward sequencing.

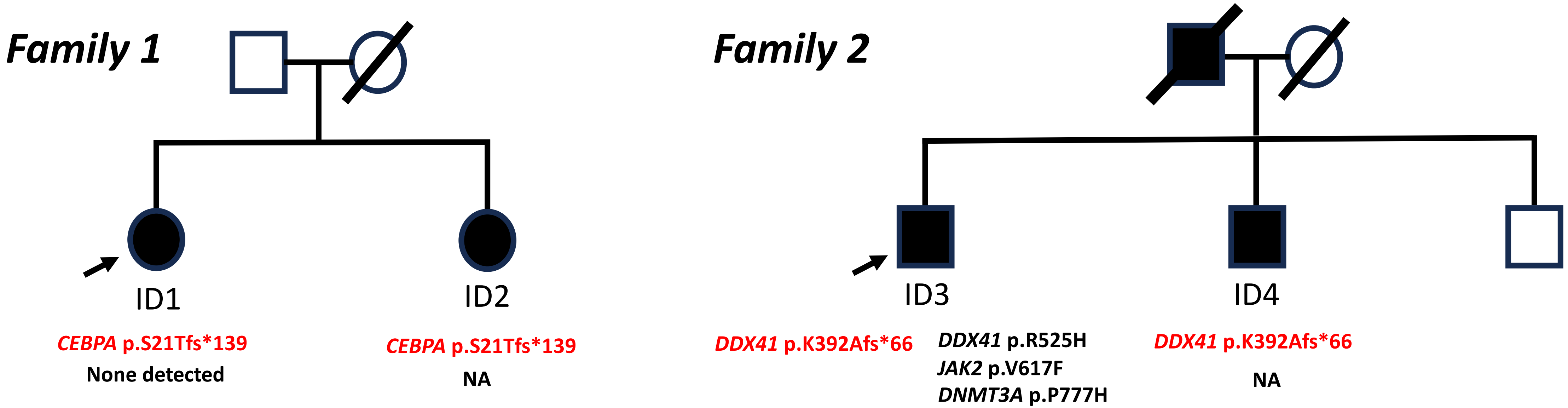

Supplementary Figure 3. Family trees of Family 1 and 2 of our cohort, showing the germline mutations (red font) as well as the somatic mutations (black font) identified. Squares indicate male family members, circles female members. Unaffected individuals are indicated with open symbols, affected individuals are shown with filled symbols, and symbols with a slash denote deceased individuals. Arrow indicates the proband. NA, not available.

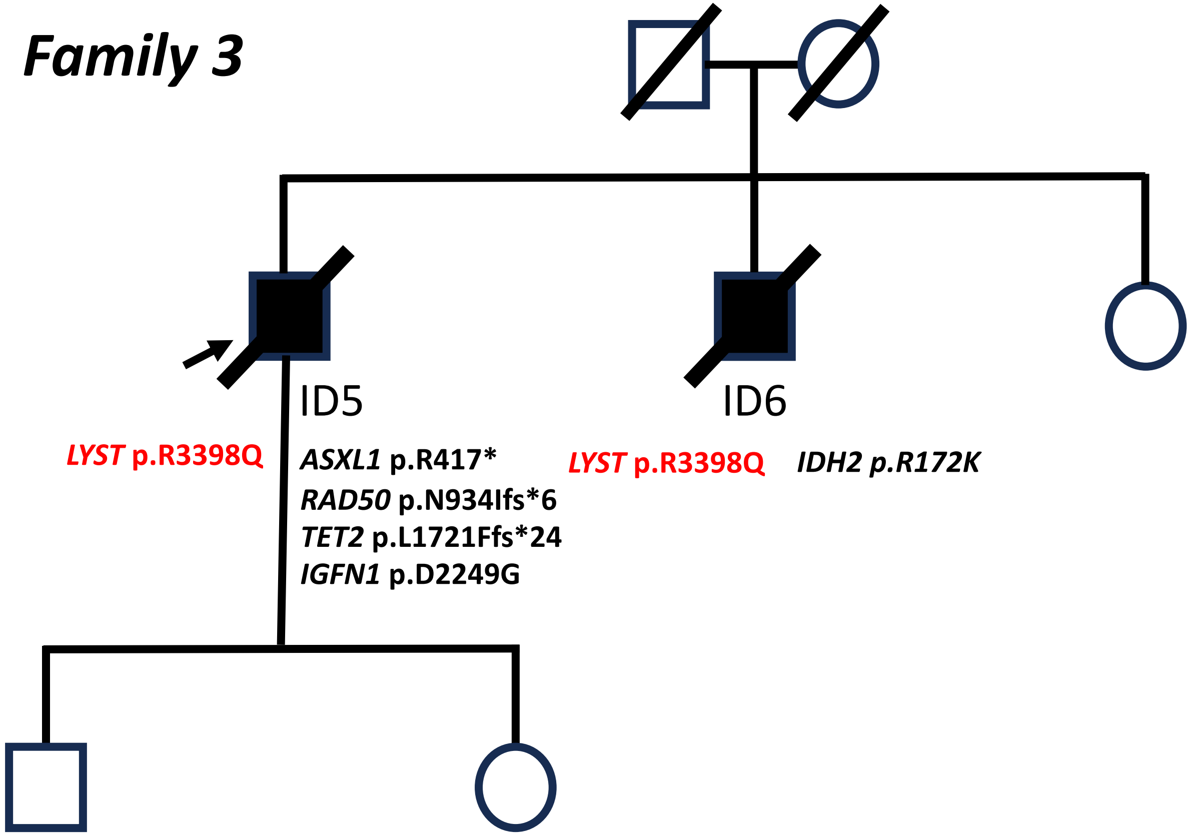

Supplementary Figure 4. Family tree of Family 3 of our cohort, showing the germline mutations (red font) as well as the somatic mutations (black font) identified. Squares indicate male family members, circles female members. Unaffected individuals are indicated with open symbols, affected individuals are shown with filled symbols, and symbols with a slash denote deceased individuals. Arrow indicates the proband.

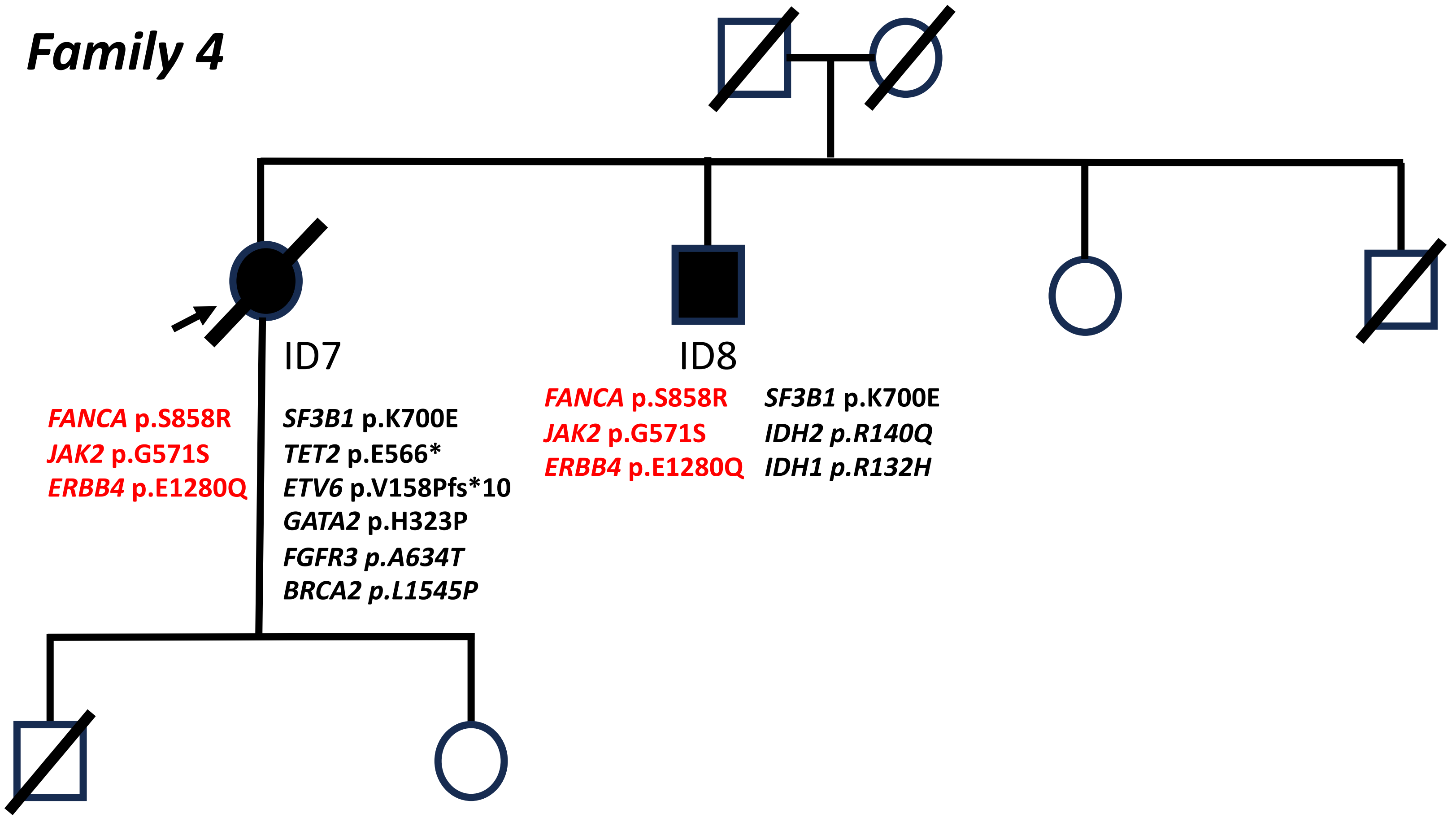

Supplementary Figure 5. Family tree of Family 4 of our cohort, showing the germline mutations (red font) as well as the somatic mutations (black font) identified. Squares indicate male family members, circles female members. Unaffected individuals are indicated with open symbols, affected individuals are shown with filled symbols, and symbols with a slash denote deceased individuals. Arrow indicates the proband.
