# Supplementary material for: Identification of Novel Potential Predisposing Variants in Familial Acute Myeloid Leukemia
Source: Cancer Rep (Hoboken). 2024 Aug 8;7(8):e2141. doi: 10.1002/cnr2.2141 (PMC11310090; doi:10.1002/cnr2.2141)
Supplement: Supplementary file 2 — Table S1. [file CNR2-7-e2141-s001.docx]

**Supplementary Table 1. List of genes and pharmacogenomics single nucleotide polymorphisms (SNPs) included in our custom Myelo Panel.**

| *ABCB7* | *CCND1* | *ERCC2* | *GNA11* | *MAPK8* | *PARN* | *REV7* | *SMARCB1* |
| --- | --- | --- | --- | --- | --- | --- | --- |
| *ABL1* | *CCND2* | *ERCC3* | *GNAQ* | *MASTL* | *PAX5* | *RFWD3* | *SMC1A* |
| *ABL2* | *CCND3* | *ERCC4* | *GP1BA* | *MDM2* | *PDGFB* | *RICTOR* | *SMC3* |
| *ACBD5* | *CCNE1* | *ERCC6L2* | *HAX1* | *MEF2B* | *PDGFRA* | *ROS1* | *SMO* |
| *ACD* | *CDAN1* | *ESR1* | *HDAC9* | *MEF2BNB-MEF2B* | *PDGFRB* | *RPL11* | *SMOX* |
| *AK2* | *CDK4* | *ETV6* | *HGF* | *MET* | *PDSS2* | *RPL15* | *SRC* |
| *AKR1B1* | *CDK6* | *EZH2* | *HNRNPK* | *MGMT* | *PGF* | *RPL23* | *SRP72* |
| *AKT1* | *CDKN1B* | *FANCA* | *HOXA11* | *MIR142* | *PGR* | *RPL26* | *SRSF2* |
| *AKT2* | *CDKN2A* | *FANCB* | *HRAS* | *MITF* | *PHF6* | *RPL27* | *STAG2* |
| *AKT3* | *CDKN2B* | *FANCC* | *HSP90AA1* | *MLH1* | *PIK3C2B* | *RPL31* | *STK11* |
| *ALAS2* | *CDKN2C* | *FANCD2* | *ID3* | *MMP2* | *PIK3CA* | *RPL35A* | *STK4* |
| *ALK* | *CEBPA* | *FANCE* | *IDH1* | *MPL* | *PIK3CB* | *RPL36* | *SYK* |
| *ANKRD26* | *CHEK2* | *FANCF* | *IDH2* | *MSH2* | *PIK3CD* | *RPL4* | *TAOK1* |
| *AP3B1* | *CHEK2 (CHK2)* | *FANCG* | *IGF1R* | *MSH6* | *PIK3R1* | *RPL5* | *TAOK2* |
| *APC* | *CREBBP* | *FANCI* | *IGF2* | *MST1R* | *PIK3R2* | *RPS10* | *TAZ* |
| *AR* | *CSF1R* | *FANCL* | *IKZF1* | *MTOR* | *PIP5K1A* | *RPS15* | *TCIRG1* |
| *ARAF* | *CSF3R* | *FANCM* | *JAGN1* | *MXRA5* | *PLCG2* | *RPS17* | *TEK* |
| *ASXL1* | *CTC1* | *FANCP* | *JAK1* | *MYC* | *PML* | *RPS19* | *TERC* |
| *ATG2B/GSKIP* | *CTNNB1* | *FANCQ* | *JAK2* | *MYCN* | *PMS2* | *RPS24* | *TERT* |
| *ATM* | *CXCR4* | *FBXW7* | *JAK3* | *MYD88* | *POT1* | *RPS26* | *TET2* |
| *ATR* | *DDR1* | *FGF3* | *KDR* | *MYH9* | *PRKCZ* | *RPS27* | *TINF2* |
| *AURKA* | *DDR2* | *FGF4* | *KIT* | *NAF1* | *PTCH1* | *RPS27A* | *TOP2A* |
| *AURKB* | *DDX41* | *FGFR1* | *KLF1* | *NBN (NBS1)* | *PTEN* | *RPS28* | *TP53* |
| *AURKC* | *DKC1* | *FGFR2* | *KMT2A* | *NCOR2* | *PTPDC1* | *RPS29* | *TSC1* |
| *BAP1* | *DNAJC21* | *FGFR3* | *KMT2D* | *NF1* | *PTPN11* | *RPS7* | *TSC2* |
| *BCL2* | *DNMT1* | *FGFR4* | *KRAS* | *NF2* | *PUS1* | *RTEL1* | *TYK2* |
| *BCOR* | *DNMT3A* | *FH* | *LAMTOR* | *NHP2* | *RAB27A* | *RUNX1* | *U2AF1* |
| *BCR* | *DOT1L* | *FKBP5* | *LCK* | *NOP10* | *RAC1* | *SAMD9* | *UBE2T* |
| *BLM (RECQL3)* | *EGFR* | *FLCN* | *LIG4* | *NOTCH1* | *RAC2* | *SAMD9L* | *USB1* |
| *BRAF* | *ELANE* | *FLT1* | *LYN* | *NOTCH2* | *RAD21* | *SBDS* | *VEGFA* |
| *BRCA1* | *EPCAM* | *FLT3* | *LYST* | *NOTCH3* | *RAD50* | *SEC23B* | *VEGFB* |
| *BRCA2* | *EPHA1* | *FLT4* | *MAP2K1* | *NOTCH4* | *RAD51* | *SF3B1* | *VPS13B* |
| *BRIP1* | *EPHA2* | *FUS* | *MAP2K2* | *NPM1* | *RAD51C* | *SF3B2* | *VPS45* |
| *BTK* | *EPHA3* | *FYN* | *MAP2K4* | *NRAS* | *RAF1* | *SH2B3* | *WAS* |
| *C15orf41* | *EPHA4* | *G6PC3* | *MAP3K1* | *NTRK1* | *RARA* | *SLC19A2* | *WRAP53* |
| *CALR* | *EPHB2* | *GATA1* | *MAP3K11* | *NTRK2* | *RBBP6* | *SLC25A38* | *WT1* |
| *CBFB* | *ERBB2* | *GATA2* | *MAP3K4* | *NTRK3* | *RBM8A* | *SLC37A4* | *XRCC2* |
| *CBL* | *ERBB3* | *GFI1* | *MAP4K1* | *PALB2* | *RECQL* | *SLTM* | *YES1* |
| *CBX7* | *ERBB4* | *GLRX5* | *MAPK1* | *PAPD5* | *RET* | *SLX4* | *rs1045642* |
| *rs2032582* | *rs9024* | *rs11572080* | *rs121434569* | *rs1138272* | *rs12948783* | *rs10981694* | *rs6755571* |
| *rs1128503* | *rs8133052* | *rs3892097* | *rs11568315* | *rs9274407* | *rs1042858* | *rs4795541* | *rs6431558* |
| *rs246240* | *rs9344* | *rs2070673* | *rs2227983* | *rs12654264* | *rs9937* | *rs4149015* | *rs3832043* |
| *rs3740066* | *rs2072671* | *rs2740574* | *rs712829* | *rs17583889* | *rs2898950* | *rs4149056* | *rs1801019* |
| *rs717620* | *rs532545* | *rs35599367* | *rs2234922* | *rs430397* | *rs1561876* | *rs2306283* | *rs25487* |
| *rs2273697* | *rs602950* | *rs776746* | *rs1136201* | *rs6313* | *rs1042919* | *rs11045585* | *rs1382368* |
| *rs1051640* | *rs60369023* | *rs17574269* | *rs11615* | *rs2075252* | *rs1130609* | *rs4880* | *rs8060157* |
| *rs9561778* | *rs3215400* | *rs2297595* | *rs3212986* | *rs1801133* | *rs5030743* | *rs2302948* | *rs20572* |
| *rs16950650* | *rs4646316* | *rs1801159* | *rs13181* | *rs1801131* | *rs1265138* | *rs10426377* | *rs4244285* |
| *rs2231137* | *rs9332377* | *rs17376848* | *rs2207396* | *rs1799983* | *rs1979277* | *rs1042522* | *rs121434568* |
| *rs2494752* | *rs4646* | *rs67376798* | *rs396991* | *rs2070744* | *rs9514091* | *rs1142345* | *rs518329* |
| *rs7921977* | *rs1048943* | *rs1801265* | *rs2297480* | *rs1800566* | *rs1051266* | *rs1800460* | *rs1695* |
| *rs2227310* | *rs1056836* | *rs55886062* | *rs1736557* | *rs1143684* | *rs11231825* | *rs12201199* | *rs885004* |
| *rs12415607* | *rs3745274* | *rs1801158* | *rs12613732* | *rs10932125* | *rs12210538* | *rs34489327* | *rs4148323* |
| *rs4353229* | *rs3211371* | *rs9981861* | *rs9679162* | *rs17626122* | *rs714368* | *rs34743033* |  |
| *rs1127687* | *rs12721655* | *rs4444903* | *rs1806201* | *rs870995* | *rs316019* | *rs8175347* |  |
